# BAI1-Associated Protein 2-Like 1 (BAIAP2L1) Is a Potential Biomarker in Ovarian Cancer

**DOI:** 10.1371/journal.pone.0133081

**Published:** 2015-07-29

**Authors:** Angel Chao, Chia-Lung Tsai, Shih-Ming Jung, Wei-Chi Chuang, Chieh Kao, An Hsu, Shun-Hua Chen, Chiao-Yun Lin, Yi-Chao Lee, Yun-Shien Lee, Tzu-Hao Wang, Hsin-Shih Wang, Chyong-Huey Lai

**Affiliations:** 1 Department of Obstetrics and Gynecology, Chang Gung Memorial Hospital and Chang Gung University, Taoyuan, Taiwan; 2 Genomic Medicine Research Core Laboratory, Chang Gung Memorial Hospital, Taoyuan, Taiwan; 3 Department of Clinical Pathology, Chang Gung Memorial Hospital and Chang Gung University, Taoyuan, Taiwan; 4 Graduate Institute of Biomedical Science, School of Medicine, Chang Gung University, Taoyuan, Taiwan; 5 College of Medical Science and Technology, Taipei Medical University, Taipei, Taiwan; 6 Department of Biotechnology, Ming-Chuan University, Taoyuan, Taiwan; 7 School of Traditional Chinese Medicine, College of Medicine, Chang Gung University, Taoyuan, Taiwan; The University of Hong Kong, HONG KONG

## Abstract

**B**rain-specific **a**ngiogenesis **i**nhibitor 1 (BAI1)-**a**ssociated **p**rotein 2-like 1 (BAIAP2L1), also known as insulin receptor tyrosine kinase substrate (IRTKS), is involved in plasma membrane protrusion and actin formation during cell morphogenesis and migration. BAIAP2L1 is recently reported to promote cell proliferation through activation of the EGFR-ERK pathway in hepatocellular carcinoma. In this study, we report the first comprehensive study of BAIAP2L1 upregulation in human ovarian cancer. Upregulation of BAIAP2L1 in ovarian tumors was first found during RNA screening and confirmed by immunohistochemical studies on ovarian cancers and other cancer types. Significant upregulation of BAIAP2L1 in ovarian cancer was validated by analyzing multiple independent cohorts in publicly available data sets. Furthermore, BAIAP2L1 protein expression in metastatic lesions was higher than the corresponding primary tumors. Functional assays in ovarian cancer cells revealed that BAIAP2L1 is involved in promoting cell proliferation and avoiding apoptosis. In conclusion, results of this study not only indicate that BAIAP2L1 can be used as a biomarker for human ovarian cancer but also reveal its role in cancer biology. Further elucidation of the role of BAIAP2L1 in context of the insulin receptor signaling pathways of cancer cells is warranted for developing cancer therapeutics by targeting cancer-specific metabolism.

## Introduction

Epithelial ovarian cancer is the leading cause of death in gynecological malignancies [1]. It is, along with peritoneal carcinoma and fallopian tube carcinoma, the ninth most common death in women in Taiwan [2]. The overall survival of ovarian cancer remains poor despite improvements in the treatment of ovarian cancer [3,4]. The typical late diagnosis of ovarian cancer, when the disease has already spread beyond the pelvis at the time of clinical presentation, is part of the reason for its poor outcome [5]. Immunological detection of serum cancer antigen CA125 [6] has been integrated into standard practice for the diagnosis of ovarian masses [7]. CA125 is also useful for monitoring response and facilitating surveillance for patients with ovarian cancer [8]. However, the level of CA125, with a sensitivity of 80%, is not elevated in all ovarian tumors, and the specificity can be affected by other malignancies, physiological conditions, and endometriosis [9]. Therefore, the identification of additional markers is urgently needed to complement CA125 for early detection, treatment evaluation, and assessing prognosis of patients with ovarian cancer.

BAIAP2L1/IRTKS (Gene ID 55971) is located on chromosome 7q21.3-q22.1. BAIAP2L1 encodes a 511 amino-acid protein with molecular weight approximately 57 kD [10,11]. BAIAP2L1, along with BAIAP2 (IRSp53), BAIAP2L2 (FLJ22582), belong to the IRSp53 family, and all of them share the IMD (IRSp53 and Missing-in metastasis domain) and the SH3 domain [12]. The IMD domain belongs to the larger family of the Bin-Amphipsin-Rev167 (BAR) domain [12], and the BAR domain forms a crescent-shaped dimmer that can bind highly curved, negatively charged membrane [13]. In addition, the IMD has actin filament-binding ability [12]. The SH3 domain is known for multiple protein-protein interactions [14]. Therefore, the members of the IRSp53 family are viewed as scaffold proteins or adaptors that help the assembly of protein complexes to the actin filament or to the membrane with a specific curvature.

BAIAP2L1 can modulate clusters of short actin bundles during cell movement [15]. In enterohemorrhagic *Escherichia coli*, many of the reports on BAIAP2L1 pertain its similarity to BAIAP2 in actin-rich membrane protrusions, called pedestals [16,17,18,19]. In mammalian cells, BAIAP2L1 has also been linked to Src-dependent cell migration [14,19,20] and MDM2-mediated p53 ubiquitination [21]. In tumorigenesis, BAIAP2L1 has been reported to promote cell proliferation through activation of the EGFR-ERK pathway in hepatocellular carcinoma [22]. Oncogenic FGF receptor 3 (FGFR3)-BAIAP2L1 fusion gene was identified in bladder cancers [23] and lung cancers [24], and its identification may aid in selecting patients for FGFR-targeted therapy. To date, the role of BAIAP2L1 in ovarian cancer has not been defined.

We report here the first comprehensive study of BAIAP2L1 upregulation in ovarian cancers. Upregulation of BAIAP2L1 in ovarian tumors was first revealed on Clontech Cancer Profiling Array and confirmed with immunohistochemical studies. Public domain datasets were used to validate that the expression of BAIAP2L1 is significantly higher in ovarian cancer than normal tissues and other types of cancer. Functional assays in ovarian cancer cells indicate that BAIAP2L1 is involved in promoting cell proliferation and avoiding apoptosis. These results suggest that the upregulation of BAIAP2L1 can be used as a potential biomarker in ovarian cancer.

## Material and Methods

### Microarray analysis for differentially expressed genes

Oligonucleotide microarrays containing 18861 probes (Compugen Inc., Tel Aviv, Israel) were used to analyze differentially expressed genes between the RNA of human normal ovary (catalog number CR0856, Clontech, CA, USA) and human ovarian tumor (catalog number 64011–1, Clontech, Palo Alto, CA). Procedures of CyDye labeling and scanning were similar to that were previously reported [25]. Among differentially expressed genes between the normal ovary and ovarian tumor, BAIAP2L1 was selected to be further validated. Cancer Profiling Array II membranes (catalog #7847–1, Clontech, Palo Alto, CA) were hybridized with the radiolabeled cDNA probe for BAIAP2L1. The array contains 154 samples of different types of cancer from breast, ovary, rectum, stomach, lung, kidney, bladder, vulva, prostate, uterus, cervix, thyroid gland, testis, skin, small intestine, pancreas, trachea, and liver (S1 Fig).

### Immunohistochemical analysis of clinical tumor tissues

Formalin-fixed paraffin-embedded (FFPE) tumor tissues of ovarian cancer were obtained from Chang Gung Memorial Hospital. Clinical information of the patients was archived in the databank of the Gynecologic Cancer Research Center, Chang Gung Memorial Hospital, Taiwan. The study was approved by the Institutional Review Board of the Chang Gung Memorial Hospital (**IRB No.95-1328B**). Written informed consent from the donor or the next of kin was obtained for the use of this sample in research. Commercially available tissue arrays included: FDA805-1and 805–2 (S1 Tables) (US Biomax. Inc, Rockville, MD, USA), BC 111109, BC 111110, and OV8011-2-BX (S2 Tables) (US Biomax. Inc, Rockville, MD, USA).

FFPE tissue slides (ovarian cancer and normal ovary, each 4-mm thick) were deparaffinized in xylene and rehydrated in decreasing concentrations of ethanol. The sections were immunostained with an anti- BAIAP2L1 antibody at 1:1000 dilution by using the Bond-Max automated immunohistochemical stainer (Leica, Germany). The overall immunohistochemical score (histoscore) in this study was the percentage of positive cells multiplied by its staining intensity (0 = negative, 1 = weak, 2 = moderate, 3 = strong), and ranged from 0 to 300 (100% multiplied by 3) [26,27].

### Culture and treatment of cell lines

Human ovarian cancer cell lines (TOV112D, TOV-21G, SKOV3, MDAH2774), lung fibroblast cells (HFL1), and normal aorta smooth muscle cells (T/G HA-VSMC) were obtained from the American Type Culture Collection (Manassas, VA, USA). TOV112D, TOV-21G, SKOV3, MDAH2774, and HFL1 were cultured in DMEM/F12 with 10% fetal bovine serum and appropriate amounts of penicillin and streptomycin at 37°C with 5% CO2. T/G HA-VSMC cells were cultured in DMEM/F12 with 10% fetal bovine serum, 0.05% mg/ml ascorbic acid, 0.01 mg/ml insulin, 0.01 mg/ml transferrin, 10 ng/ml sodium selenite, and 0.03 mg/ml endothelial cell growth supplement. For apoptosis analysis, cells transfected with BAIAP2L1 siRNA were either irradiated with UV (100 J/M^2^) or treated with 10 μM cisplatin (Fresenius Kabi, NC, USA) for 24 hrs before analysis.

### Transfection of small interfering (si) RNA

MDAH2774 and SKOV3 (8000 and 4000 cells, respectively, in 96-well plates) were transfected with 1 picomole of small interfering-BAIAP2L1 (CCCGAATTCACAAAGGGTAAATAAT, Invitrogen, Carlsbad, CA) in Lipofectamine RNAimax (Invitrogen, Carlsbad, CA) according to the manufacturer’s protocol. After 48 h of transfection, suppression of targeted genes was confirmed by western blot analyses.

### RNA extraction and real-time Q-PCR

Total RNA was isolated with TRIzol reagent (Invitrogen, Carlsbad, CA). For RT-QPCR, first strand cDNA was synthesized with an oligo-T primer using superscript III first strand synthesis kit (Invitrogen, Carlsbad, CA). The expression level of BAIAP2L1 (forward primer: CACCCGACTACTTGGAATGCT, reverse primer: CGTTGGCATCGTTAGGAGC) were quantified using SYBR Green assay (Applied Biosystems). Glyceraldehyde-3-phosphate dehydrogenase (GAPDH) expression (forward primer: 5’-GGTATCGTGGAAGGACTCATGAC-3’, reverse primer: 5’-ATGCCAGTGAGCTTCCCGT-3’) was used as internal control. The thermocycles were as follow: initial denaturation at 95°C for 10 min, followed by 45 cycles of 95°C for 15 s and 60°C for 1 min. The reactions were performed in the ABI PRISM 7900 HT instrument (Applied Biosystems). Calculations were done with the mean cycle of threshold (Ct) value for each duplicate measurements.

### Western blot analysis

Cells were lysed in ice-cold radioimmunoprecipitation assay lysis buffer [1% Triton X-100, 1% NP-40, 0.1% SDS, 0.5% DOC, 20 mM Tris-hydroxymethyl-aminomethane (Tris-HCl, pH 7.4), 150 mM NaCl, protease inhibitors (Sigma, St. Louis, MO, USA), and phosphatase inhibitors (Sigma, St Louis, MO, USA)] for 30 minutes. Following electrophoretic separation on 13% sodium dodecyl sulfate-polyacrylamide gels, the proteins on gels were transferred to nitrocellulose membranes (Amersham Pharmacia Biotech, Uppsala, Sweden). Protein samples were analyzed using anti-BAIAP2L1 (Abnova, Taipei, Taiwan), anti-cleaved caspase 3 (Cell Signaling Technology, Danvers, MA, USA), anti-PARP (Cell Signaling Technology, Danvers, MA, USA) and anti-actin (Santa Cruz Biotechnology, Santa Cruz, CA) as primary antibodies, and corresponding horseradish peroxidase-conjugated secondary antibodies (Santa Cruz Biotechnology, Santa Cruz, CA). Labeled proteins were subsequently detected by enhanced chemiluminescence (ECL, Millipore, and Bradford, MA, USA). For each sample, band intensities were normalized to β-actin.

### Cell viability assay

After knockdown of endogenous BAIAP2L1 expression with siRNA for 48 h, cell proliferation activity was measured with 3-(4,5-Dimethylthiazol-2-yl)-2,5 Diphenyltetrazolium (MTT) method (Sigma, M5655-1G). MDAH2774 and SKOV3 cells were plated at 8000 and 4000 cells/well, respectively, in 96-well plates. Working concentration of MTT solution was 1 mg/ml. Optical density was measured using VICTOR2 Scanning multi-well spectrophotometer with the absorbance at 570 nm. For BrdU assay, the BAIAP2L1 knockdown ovarian cancer cells were incubated with BrdU for 6 h. DNA synthesis activity was assayed using BrdU ELISA kit (Roche Applied Science) as previously described [26].

### Detection of gene fusion between FGFR and BAIAP2L1

The total RNA was extracted from 8 freshly frozen ovarian cancer tissues with protocols that were previously reported [28]. Detection of FGFR3-BAIAP2L1 fusion gene was performed with KAPA2G fast PCR kits (Kapabiosystem, Wilmington, MA) in the following conditions: 95°C for 3 min, followed by 35 cycles of 95°C for 30 sec, 53°C for 30 sec and 72°C for 30 sec, and ended at 72°C for 10 min. The PCR primers were previously reported [24]. The RNA of bladder cancer SW780 cells was used as a positive control for detecting the FGFR3-BAIAP2L1 fusion gene [23].

### Data analysis of microarrays in publically available databases

Data with the series number GSE14407 [29], GSE2109, and GSE36133 [30] were retrieved from Gene Expression Omnibus (GEO, http://www.ncbi.nlm.nih.gov/projects/geo/). Expression analysis was performed using Affymetrix Expression Console (Affymetrix, Santa Clara, CA).

### Data analysis

The results of *in vitro* studies are derived from at least three independent experiments and analyzed with two-tailed t-tests. The differences in the histoscores between two groups were compared using the student T test. Two-tailed P-values < 0.05 were considered significant. Statistical analyses were performed with the SPSS statistical software, version 17.0 (SPSS Inc, Chicago, IL, USA).

## Results

### Ovarian cancers express higher levels of BAIAP2L1 than other cancers

**Fig 1:**
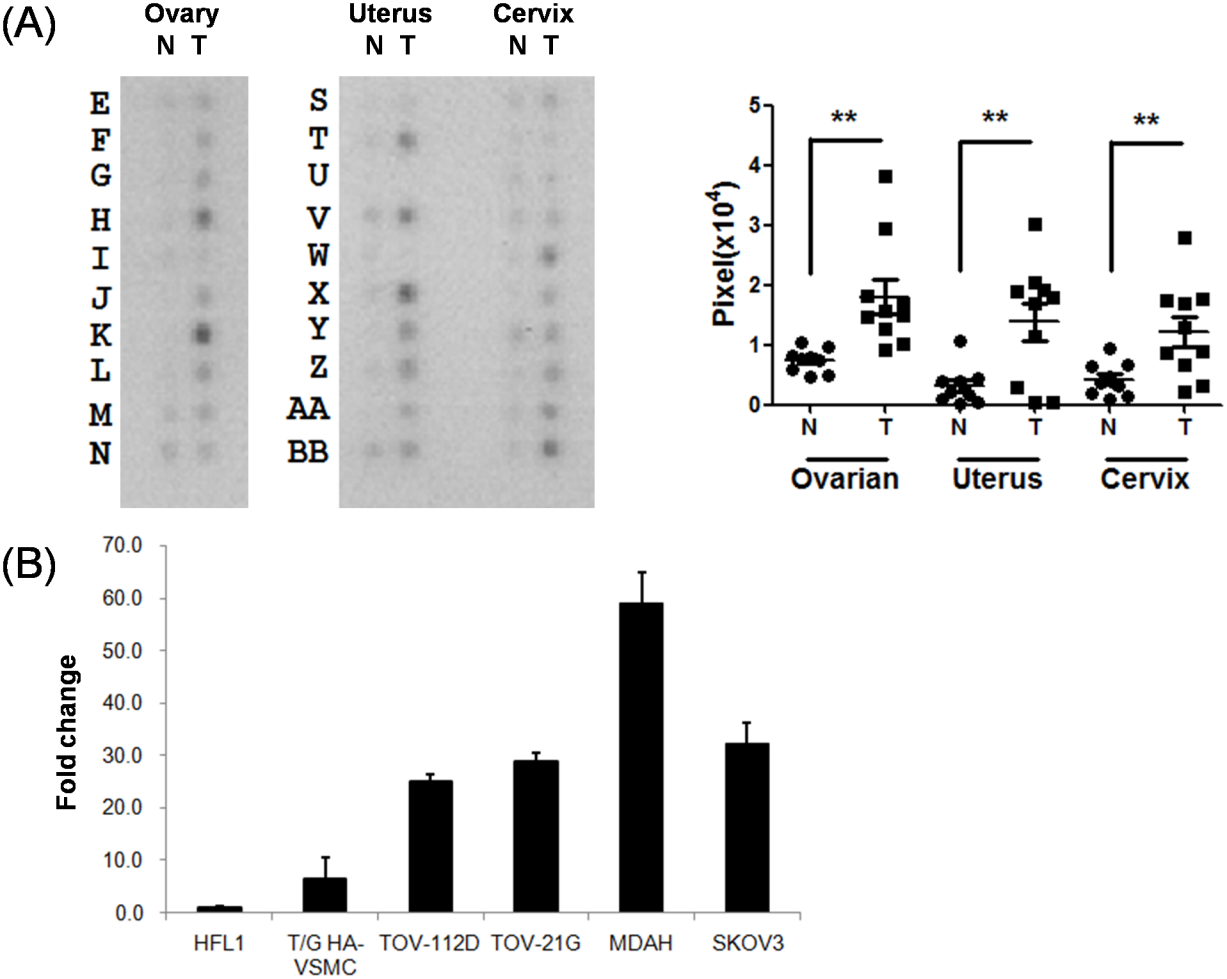
The mRNA profiles of BAIAP2L1 on tissues and cell lines. (A) The Cancer Profiling Array II (BD Clontech) of cDNA derived from normal tissues (N) and tumor tissues (T) of ovary, uterus, and cervix. Quantitation of the mRNA of tumor and normal tissues of different sites are shown at the right panel. BAIAP2L1 expression in tumors was significantly higher than that in normal tissues (** P<0.01). (B) BAIAP2L1 RNA expression in ovarian cancer cell lines was higher than normal cell lines. Ovarian cancer lines included serous type (SKOV3), endometrioid type (TOV112D, MDAH2774), and clear cell type (TOV21G). HFL1 is human normal lung fibroblast cell, and T/G HA-VSMC is human normal aorta smooth muscle cells. *Abbreviations*: T, tumor; N, normal.

**Fig 2:**
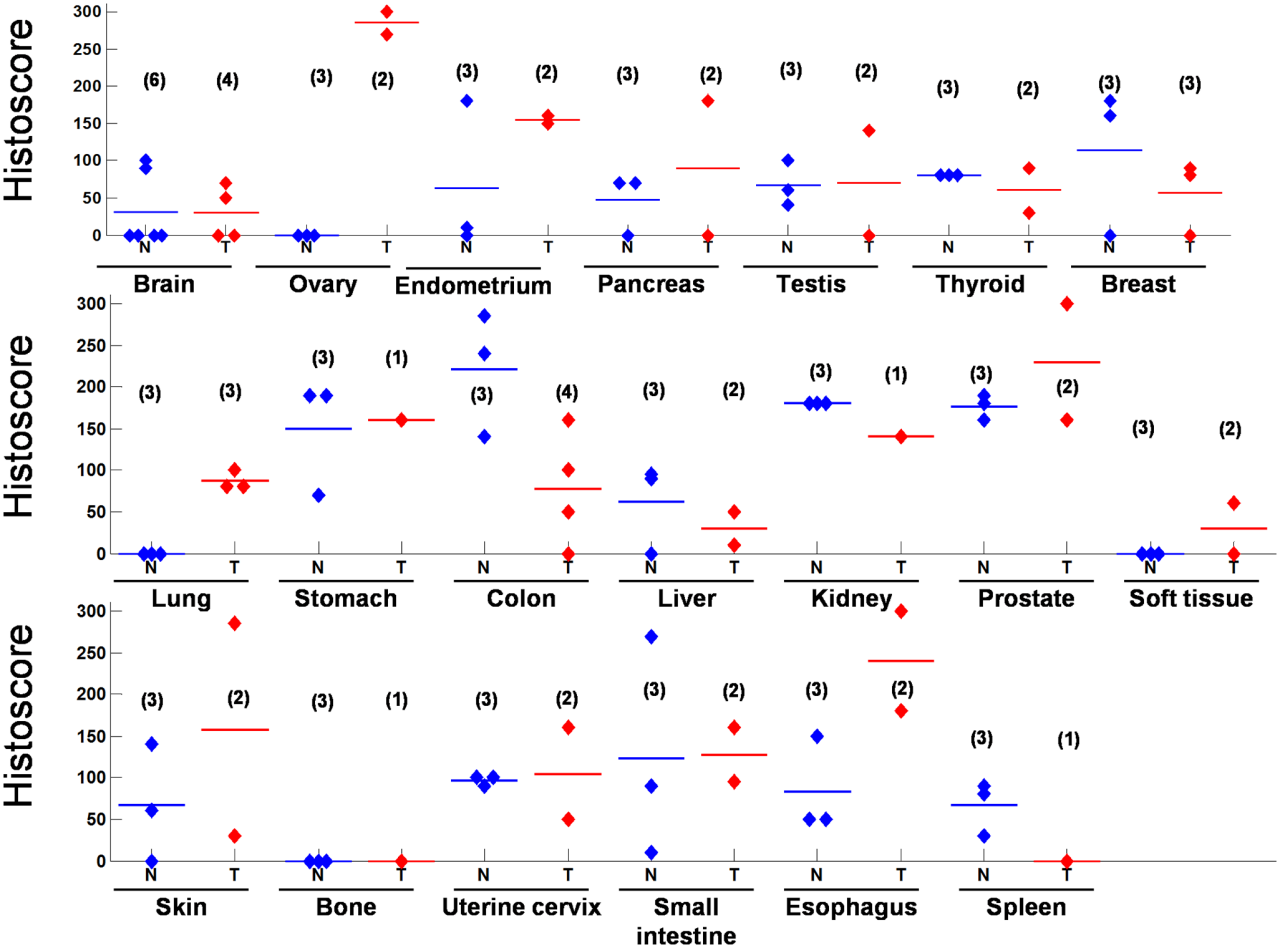
BAIAP2L1 histoscores in multiple human organs and corresponding tumors. Results were derived from commercially available tissue arrays FDA805-1and 805–2, containing normal organs and corresponding tumors (S2 Tables). Number in parentheses denotes the number of cases in the tissue arrays. The overall immunohistochemical score (histoscore) was the percentage of positive cells multiplied by its staining intensity (0 = negative, 1 = weak, 2 = moderate, 3 = strong), and ranged from 0 to 300 (100% multiplied by 3).

The 10 ovarian tumors specimens studied comprised of 6 malignant cancers, 2 borderline serous cystadenoma (coordinate #E and N), and 2 leiomyoma (coordinates #F and J) (Fig 1A). Other gynecologic cancer and normal tissues of uterine and cervical origins were also determined. In all three tissue types, BAIAP2L1 expression levels in tumor tissues were significantly higher than normal tissues. Similarly, mRNA expression of BAIAP2L1 in ovarian cancer cell lines was higher than normal cell lines (Fig 1B). In agreement with RNA expression results, BAIAP2L1 protein expression is higher in ovarian cancer than in normal tissues, as shown in FDA805-1and 805–2 tissue arrays (Fig 2).

### Metastasized ovarian cancer tissues express higher BAIAP2L1 than primary cancer

**Fig 3:**
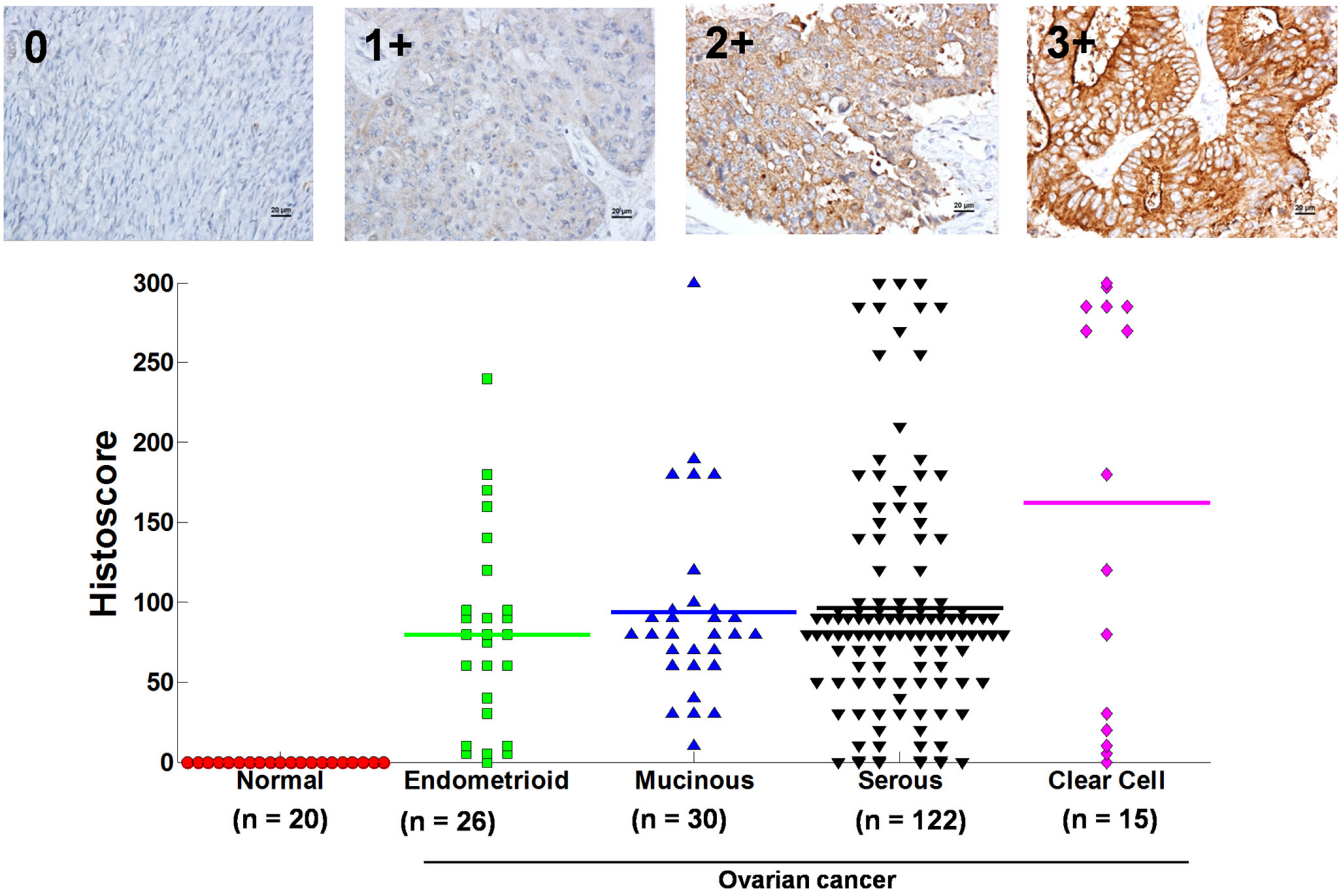
BAIAP2L1 histoscores in different subtypes of ovarian cancer. Immunohistochemical studies were performed on ovarian cancer tissues (n = 193) and normal controls (n = 20). Upper panel indicates the immunohistochemical staining intensity (0 = negative, 1 = weak, 2 = moderate, 3 = strong). The overall immunohistochemical score (histoscore) was the percentage of positive cells multiplied by its staining intensity, and ranged from 0 to 300 (100% multiplied by 3).

**Fig 4:**
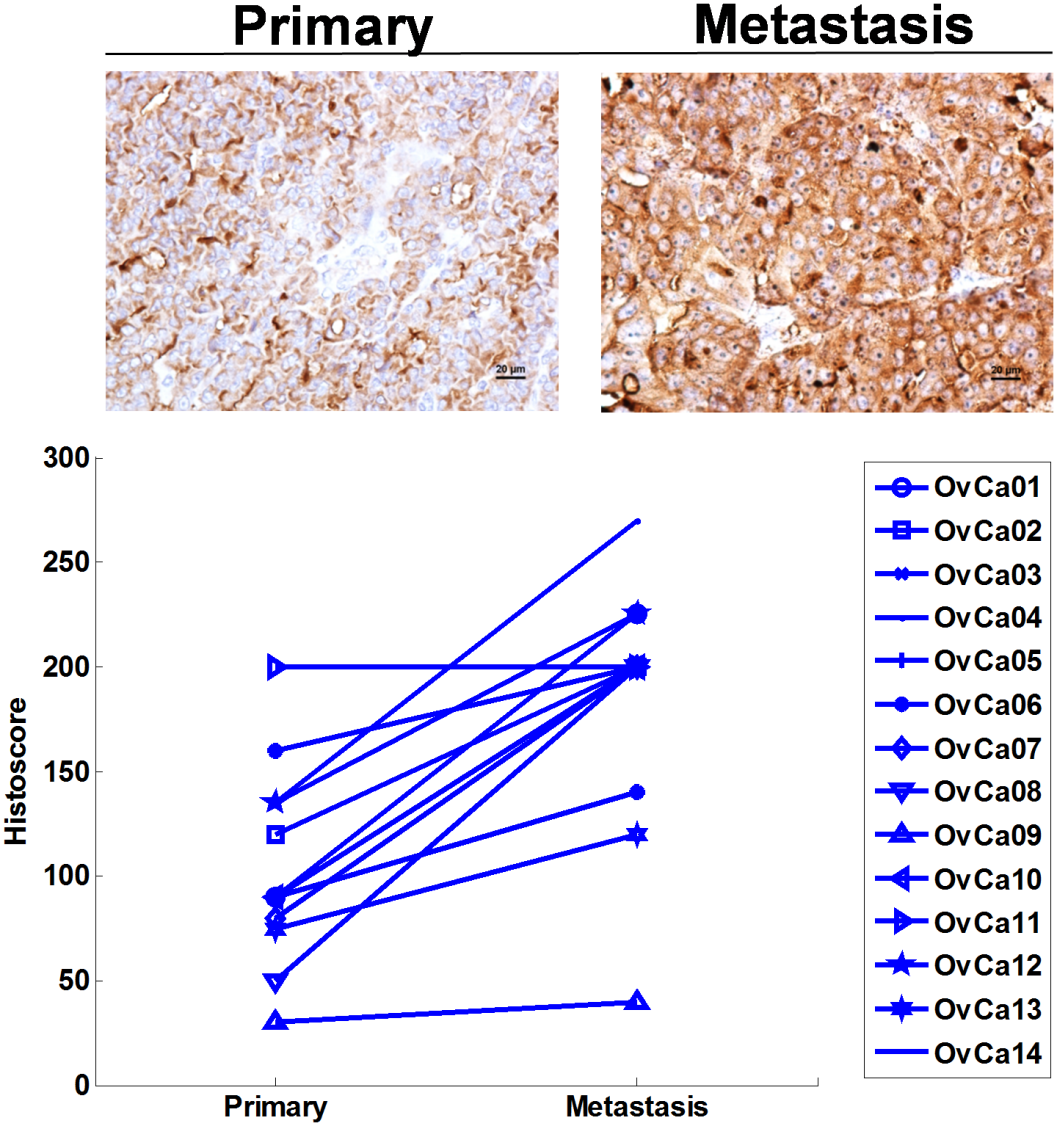
BAIAP2L1 levels are significantly higher in metastatic sites than primary ovarian cancer tissues (n = 14). The upper panel shows BAIAP2L1 expression in a representative pair of primary ovarian cancer and its metastatic site.

Protein levels of BAIAP2L1 in 193 ovarian cancer tissues, containing serous, endometrioid, clear cell, and mucinous cell types were analyzed (Fig 3). Expression levels of BAIAP2L1 were not different among various cell types of ovarian cancer, stage and grade of each histologic subtype, but each type of ovarian cancer expressed significantly higher BAIAP2L1 than normal ovarian tissues (Fig 3). Expression of BAIAP2L1 was further analyzed in 14 paired samples of primary ovarian cancers and their corresponding metastatic sites (S3 Tables). The metastatic sites include omentum (n = 7), lymph node (n = 3), parametrium (n = 1), small bowel (n = 1), cecum (n = 1), and brain (n = 1). Twelve of the 14 pairs had higher BAIAP2L1 expressions in the metastatic sites than in primary tumors (p < 0.05) (Fig 4).

### Multiple, independent data sets validate up-regulation of BAIAP2L1 in ovarian cancer

**Fig 5:**
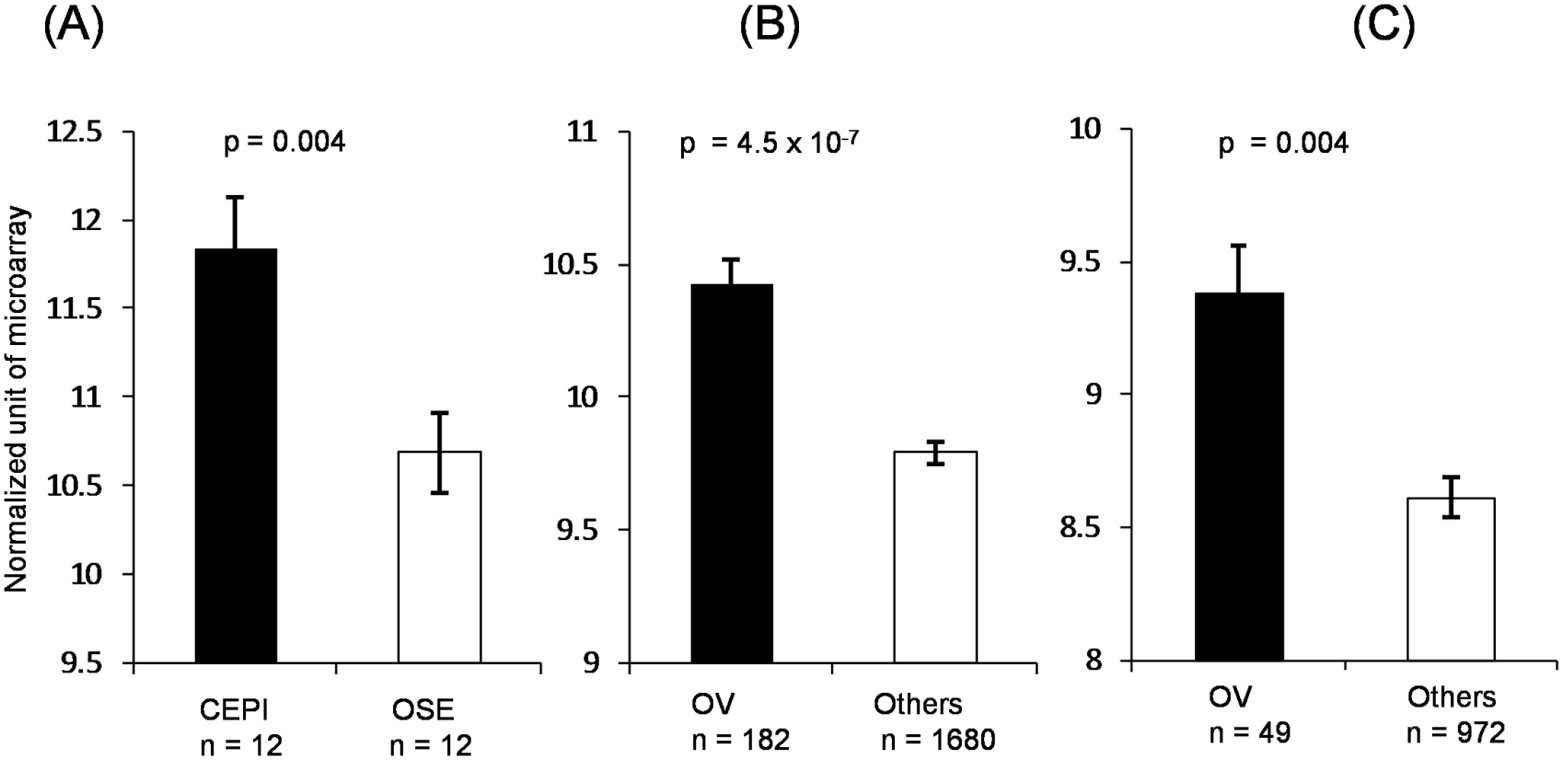
Significantly high expression of BAIAP2L1 in ovarian cancer tissues and ovarian cancer cell lines. (A) The mRNA expression levels of BAIAP2L1 in ovarian cancer epithelial cells (CEPI) isolated from12 ovarian serous adenocarcinoma and ovarian surface epithelium (OSE) from 12 normal human ovaries. Data are retrieved from GSE14407. (B) The expression levels of BAIAP2L1 in tissues of ovarian cancer (OV, n = 182) and cancer of other primary sites (n = 1680). Data are retrieved from GSE36133. (C) The expression levels of BAIAP2L1 in cell lines of ovarian cancer (OV, n = 49) and other origins (n = 972). Data are retrieved from GSE2109.

Data in GSE14407 data set [29] indicated that BAIAP2L1 mRNA expression was significantly higher in ovarian cancer epithelial cells (CEPI, 11.8 ± 0.28 shown as mean ± standard error) than in normal ovarian surface epithelia (OSE, 10.8 ± 0.22) (p = 0.004) (Fig 5A). In the tissue expression level, GSE36133 data set showed that BAIAP2L1 was significantly higher in 182 primary ovarian and fallopian tubal cancers (10.4 ± 0.09) compared to 1680 primary cancers mostly from breast, colon, rectum, endometrium, lung, and kidney (9.8 ± 0.04) (p = 4.5 x 10^−7^) (Fig 5B). GSE2109 data set [30] further revealed that BAIAP2L1 was significantly higher in 49 ovarian cancer cell lines (9.4 ± 0.17) than 972 cancer cell lines derived from other tissues (8.6 ± 0.07) (p = 0.004) (Fig 5C).

### Absence of gene fusion between FGFR and BAIAP2L1 in ovarian cancer

None of 8 tested ovarian cancer RNA contained the transcript of fusion gene between FGFR and BAIAP2L1, while the RNA of bladder cancer SW780 cells expressed transcripts of the fusion gene (S2 Fig).

### BAIAP2L1 not only promotes cell proliferation but also prevents apoptosis in ovarian cancer cells

**Fig 6:**
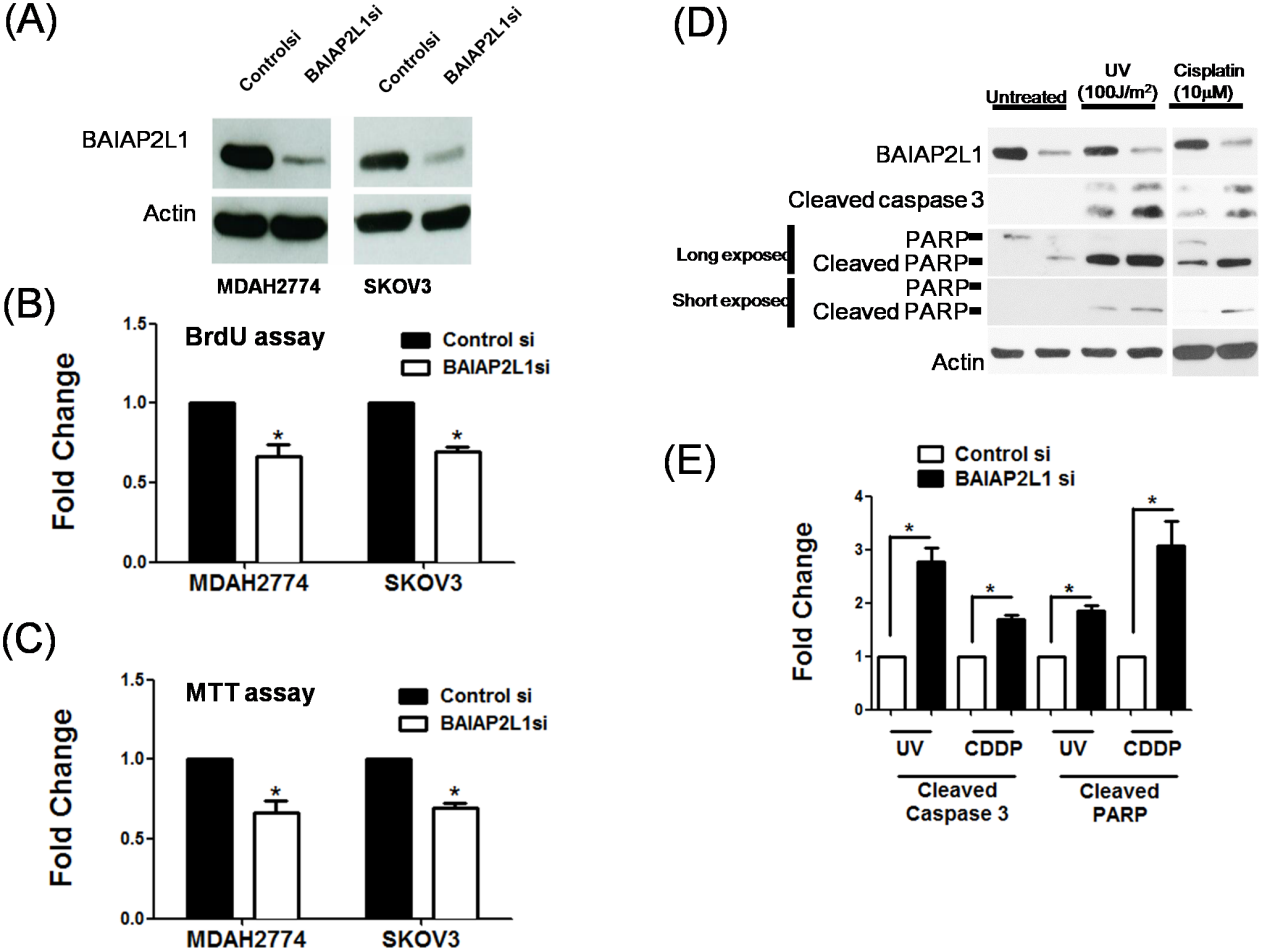
Knockdown of endogenous BAIAP2L1 inhibits cell proliferation and enhances apoptosis induced by UV and cisplatin. BAIAP2L1 in two ovarian cancer cell lines (MDAH2774 and SKOV3) were knockdowned with si-RNA (A) and analyzed with (B) BrdU incorportation assay and (C) MTT assay. (D) BAIAP2L1 knockdown in SKOV3 cells increased cleavage of caspase 3 and PARP, which were indicators of apoptosis. The cells were irradiated with UV (100 J/M^2^) or treated with 10 μM cisplatin for 24 h to induce apoptosis. To clearly visualize the differential intensities of cleaved PARP bands, two exposure times were used during chemiluminescence: long (60 sec) and short (10 sec). The actin level was used to normalize the amount of input protein. (E) Quantitative analyses of cleaved caspase 3 and PARP. Results shown are the mean ± standard error from three independent experiments. Asterisks denote statistical significance (p < 0.05).

Since BAIAP2L1 has been shown to promote cell proliferation in hepatocellular carcinoma (HCC) [22], we also tested the role of BAIAP2L1 in cell growth of ovarian cancer cells. Knockdown of BAIAP2L1 with (Fig 6A) siRNA technology inhibited cell growth in multiple ovarian cancer cell lines, shown in results of BrDU incorporation assays (Fig 6B) and MTT assays (Fig 6C).

In apoptosis pathways, pro-caspase 3 is activated and cleaved into 17-kDa and 12-kDa fragments, and then proteolytic enzyme digests downstream proteins, such as poly (ADP-ribose) polymerase (PARP) [31]. Without DNA insults (UV or cisplatin), knockdown of BAIAP2L1 alone slightly increased cleaved PARP levels (Fig 6D and 6E). After UV irradiation or cisplatin treatment for 24 h, knockdown of BAIAP2L1 increased protein level of cleaved caspase 3 and cleaved PARP. These results suggest that BAIAP2L1 may protect cells from apoptosis.

## Discussion

BAIAP2L1 expression has been shown to increase in hepatocellular carcinoma [22]. In this study, integrative database analyses of BAIAP2L1 in GSE14407 and GSE36133 cancer tissues as well as GSE2109 cell lines validate our immunohistochemical results of high BAIAP2L1 expression in ovarian cancers. These results indicate, for the first time, that BAIAP2L1 is upregulated in human ovarian cancers, accounting for increased cell proliferation and resistance to apoptosis of ovarian cancer.

Our findings that BAIAP2L1 histoscores of metastatic sites are higher than primary ovarian cancers suggest that BAIAP2L1 may contribute to tumor invasion and metastasis. These results are in agreement with the findings that BAIAP2L1 is involved in plasma membrane deformation and actin cytoskeleton remodeling, which are important for cell migration [20]. Although oncogenic FGF receptor 3 (FGFR3)-BAIAP2L1 fusion gene has been identified in bladder cancers [23] and lung cancers [24], we could not detect the transcript of fusion genes of BAIAP2L1 in this study of a limited number of specimens. The presence of FGFR-BAIAP2L1 gene fusion has been shown to translate a larger protein in other cancers [24], but we identified BAIP2L1 only as a single band of 57 kD on western blot analysis of ovarian cancer cells, ruling out the presence of FGFR-BAIAP2L1 gene fusion.

BAIAP2L1 has also been shown in the disease progression of rheumatoid arthritis, where BAIAP2L1 expression in fibroblast-like synovial cells was positively correlated with C-reactive protein (CRP), a common clinical marker for inflammation [32]. CRP levels have been shown to predict survival in patients with renal cell carcinoma, bladder cancers, and prostate cancer, and the incorporation of CRP into prognostic models for those cancers improves the models' predictive accuracy [33]. These findings collectively support that BAIAP2L1 is involved in inflammation and tumorigenesis in general [34].

The most conclusive functions of BAIAP2L1 in mammals have been recently revealed by Han’s group using a knockout mouse approach [35]. BAIAP2L1 acts as an insulin receptor (IR) adaptor that activates the IR-IRS1/2 (insulin receptor substrate 1/2)-AKT signaling pathway via stimulating tyrosine phosphorylation of IR. BAIAP2L1-deficient mice displayed insulin resistance and abnormal glucose homeostasis [35]. Furthermore, hepatic expression of BAIAP2L1 was decreased in mice and humans with type 2 diabetes, suggesting an association between BAIAP2L1 downregulation and the development of diabetes. Interestingly, the hepatic decrease of BAIAP2L1 in diabetic patients seemed to have a male-female discrepancy, which the authors postulated to be caused by insufficient sample sizes [35]. In light of a genome-wide association study where BAIAP2L1 is highly associated with circulating levels of sex hormone-binding globulin [36], a sex dependent regulation of BAIAP2L1 functions remains worthy studying.

In addition to actin-related functions of BAIAP2L1, which are mainly elucidated from bacterial studies [16,17,18,19], the discovery of BAIAP2L1 in the IR-IRS-AKT pathways may shed light to its role in cancer-specific metabolism. To provide building blocks for tumor growth, cancer cells increase fatty acid synthesis and glutamine metabolism, at the expense of using inefficient aerobic glycolysis [37]. The concentrations of glucose in solid tumors are generally lower than normal tissues [38], thus cancer cells often crave glucose and glutamine [39]. Future studies may prove that BAIAP2L1 is involved in getting more glucose entry to cancer cells, perhaps by increasing the efficiency of Glut transporters. In addition, several downstream effectors of IR include (i) MAPKs that are known to promote cell proliferation [22] and (ii) PI-3K that increases synthesis of proteins and fatty acids and prevent cancer cells from apoptosis [40].

## Conclusions

Results of this study not only indicate that BAIAP2L1 can be used as a biomarker for human ovarian cancer but also reveal its role in cancer biology. Clinical studies with large numbers of samples and quantitative assays will be necessary to validate the usefulness of BAIAP2L1 in clinical practice. Further elucidation of the role of BAIAP2L1 in context of the IR-IRS-downstream effectors pathways of cancer cells is warranted for developing cancer therapeutics targeting cancer-specific metabolism.

## Supporting Information

S1 Fig The mRNA profiles of BAIAP2L1 on tissues and cell lines. (DOCX)

S2 Fig Absence of fusion gene between FGFR3 and BAIAP2L1. (DOCX)

S1 Tables FDA tissue arrays (805–1 and 2) and histoscores of BAIAP2L1. (DOC)

S2 Tables Tissue arrays (BC 111109, BC 111110, and OV8011-2-BX) and histoscores of BAIAP2L1. (DOCX)

S3 Tables Clinical information of 14 cases of ovarian cancers and histoscores of BAIAP2L1 in primary cancer and metastasized sites. (DOCX)
