# Supplementary material for: BAI1-Associated Protein 2-Like 1 (BAIAP2L1) Is a Potential Biomarker in Ovarian Cancer
Source: PLoS One. 2015 Jul 29;10(7):e0133081. doi: 10.1371/journal.pone.0133081 (PMC4519316; doi:10.1371/journal.pone.0133081)
Supplement: S1 Fig — (DOCX) [file pone.0133081.s001.docx]

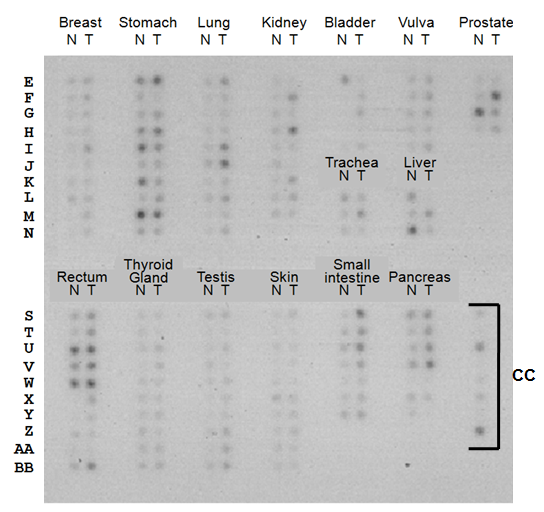


**S1 Fig. The mRNA profiles of BAIAP2L1 on tissues and cell lines.** (A) The Cancer Profiling Array II (BD Clontech) indicates cDNA of normal tissues (N) and tumor tissues (T) of different organs. Tissue sources are shown above each sample group. CC: cancer cell line DNA.
