# Supplementary material for: BAI1-Associated Protein 2-Like 1 (BAIAP2L1) Is a Potential Biomarker in Ovarian Cancer
Source: PLoS One. 2015 Jul 29;10(7):e0133081. doi: 10.1371/journal.pone.0133081 (PMC4519316; doi:10.1371/journal.pone.0133081)

**S2 Fig. Absence of fusion gene between FGFR3 and BAIAP2L1.** While reverse transcriptional PCR generated a 448-bp amplicon of the FGFR3-BAIAP2L1 fusion gene, no amplicons was derived from RNA specimens of 8 ovarian cancer tissues.


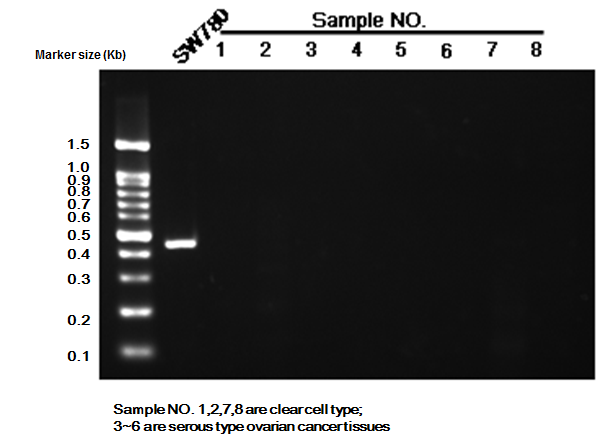

Supplement: S2 Fig — (DOCX) [file pone.0133081.s002.docx]
