# Supplementary material for: BAI1-Associated Protein 2-Like 1 (BAIAP2L1) Is a Potential Biomarker in Ovarian Cancer
Source: PLoS One. 2015 Jul 29;10(7):e0133081. doi: 10.1371/journal.pone.0133081 (PMC4519316; doi:10.1371/journal.pone.0133081)
Supplement: S1 Tables — (DOC) [file pone.0133081.s003.doc]

**S1 Tables. FDA tissue arrays (805-1 and 2) and histoscores of BAIAP2L1**

FDA 805-1

| **position** | **no.** | **sex** | **age** | **organ** | **pathol. Diagnosis** | **BAIAP2L1 histoscore** |
| --- | --- | --- | --- | --- | --- | --- |
| A1 | 1 | F | 2 | Cerebrum | Normal cerebrum tissue | 90 |
| A2 | 2 | F | 50 | Cerebrum | Normal cerebrum tissue | 100 |
| A3 | 3 | F | 42 | Cerebrum | Normal cerebrum tissue | 0 |
| A4 | 4 | F | 24 | Cerebellum | Normal cerebellum tissue | 0 |
| A5 | 5 | F | 58 | Cerebellum | Normal cerebellum tissue | 0 |
| A6 | 6 | F | 8 | Cerebellum | Normal cerebellum tissue | 0 |
| A7 | 7 | F | 14 | Adrenal gland | Normal adrenal gland tissue | 160 |
| A8 | 8 | M | 1 M | Adrenal gland | Normal adrenal gland tissue | 50 |
| A9 | 9 | M | 5 M | Adrenal gland | Normal adrenal gland tissue | 100 |
| B1 | 10 | F | 19 | Ovary | Normal ovary tissue | 0 |
| B2 | 11 | F | 20 | Ovary | Normal ovary tissue | 0 |
| B3 | 12 | F | 18 | Ovary | Normal ovary tissue | 0 |
| B4 | 13 | F | 35 | Pancreas | Normal pancreas tissue | 0 |
| B5 | 14 | M | 1 M | Pancreas | Normal pancreas tissue | 70 |
| B6 | 15 | F | 52 | Pancreas | Normal pancreas tissue | 70 |
| B7 | 16 | F | 50 | Parathyroid gland | Normal parathyroid gland tissue | 0 |
| B8 | 17 | F | 40 | Parathyroid gland | Normal parathyroid gland tissue | 70 |
| B9 | 18 | F | 40 | Parathyroid gland | Normal parathyroid gland tissue | 40 |
| C1 | 19 | F | 15 | Hypophysis | Normal hypophysis tissue | 60 |
| C2 | 20 | F | 18 | Hypophysis | Normal hypophysis tissue | 80 |
| C3 | 21 | M | 15 | Hypophysis | Normal hypophysis tissue | 30 |
| C4 | 22 | M | 30 | Testis | Normal testis tissue | 100 |
| C5 | 23 | M | 74 | Testis | Normal testis tissue | 60 |
| C6 | 24 | M | 65 | Testis | Normal testis tissue | 40 |
| C7 | 25 | F | 25 | Thyroid | Normal thyroid tissue | 80 |
| C8 | 26 | M | 37 | Thyroid | Normal thyroid tissue | 80 |
| C9 | 27 | M | 22 | Thyroid | Normal thyroid tissue | 80 |
| D1 | 28 | F | 35 | Breast | Normal breast tissue | 160 |
| D2 | 29 | F | 32 | Breast | Normal breast tissue | 180 |
| D3 | 30 | F | 33 | Breast | Normal breast tissue | 0 |
| D4 | 31 | F | 2 | Spleen | Normal spleen tissue | 30 |
| D5 | 32 | M | 35 | Spleen | Normal spleen tissue | 80 |
| D6 | 33 | M | 30 | Spleen | Normal spleen tissue | 90 |
| D7 | 34 | F | 15 | Tonsil | Normal tonsil tissue | 20 |
| D8 | 35 | M | 35 | Tonsil | Normal tonsil tissue | 0 |
| D9 | 36 | F | 18 | Tonsil | Normal tonsil tissue | 0 |
| E1 | 37 | M | 0 | Thymus gland | Normal thymus tissue | 0 |
| E2 | 38 | F | 15 | Thymus gland | Normal thymus tissue | 0 |
| E3 | 39 | F | 18 | Thymus gland | Normal thymus tissue | 0 |
| E4 | 40 | M | 56 | Bone | Normal myeloid tissue | 0 |
| E5 | 41 | F | 61 | Bone | Normal myeloid tissue | 0 |
| E6 | 42 | M | 70 | Bone | Normal myeloid tissue | 0 |
| E7 | 43 | M | 24 | Lung | Normal lung tissue | 0 |
| E8 | 44 | M | 42 | Lung | Normal lung tissue | 0 |
| E9 | 45 | M | 48 | Lung | Normal lung tissue | 0 |
| F1 | 46 | M | 56 | Heart | Normal myocardial tissue | 50 |
| F2 | 47 | F | 42 | Heart | Normal myocardial tissue | 0 |
| F3 | 48 | F | 35 | Heart | Normal myocardial tissue | 0 |
| F4 | 49 | M | 35 | Esophagus | Normal esophagus tissue | 150 |
| F5 | 50 | M | 24 | Esophagus | Normal esophagus tissue | 50 |
| F6 | 51 | F | 42 | Esophagus | Normal esophagus tissue | 50 |
| F7 | 52 | M | 48 | Stomach | Normal stomach tissue | 190 |
| F8 | 53 | M | 35 | Stomach | Normal stomach tissue | 190 |
| F9 | 54 | M | 40 | Stomach | Normal stomach tissue | 70 |
| G1 | 55 | M | 40 | Small intestine | Normal small intestine tissue | 90 |
| G2 | 56 | F | 38 | Small intestine | Normal small intestine tissue | 10 |
| G3 | 57 | M | 35 | Small intestine | Normal small intestine tissue | 270 |
| G4 | 58 | M | 62 | Colon | Normal colon tissue | 285 |
| G5 | 59 | F | 42 | Colon | Normal colon tissue | 140 |
| G6 | 60 | M | 35 | Colon | Normal colon tissue (smooth muscle tissue) | 240 |
| G7 | 61 | M | 40 | Liver | Normal liver tissue | 90 |
| G8 | 62 | F | 35 | Liver | Normal liver tissue | 0 |
| G9 | 63 | M | 35 | Liver | Normal liver tissue | 95 |
| H1 | 64 | M | 50 | Salivary gland | Normal salivary gland tissue | 40 |
| H2 | 65 | M | 77 | Salivary gland | Normal salivary gland tissue | 80 |
| H3 | 66 | M | 28 | Salivary gland | Normal salivary gland tissue | 95 |
| H4 | 67 | F | 14 | Kidney | Normal kidney tissue | 180 |
| H5 | 68 | M | 48 | Kidney | Normal kidney tissue | 180 |
| H6 | 69 | F | 50 | Kidney | Normal kidney tissue | 180 |
| H7 | 70 | M | 27 | Prostate | Normal prostate tissue | 190 |
| H8 | 71 | M | 25 | Prostate | Normal prostate tissue | 180 |
| H9 | 72 | M | 28 | Prostate | Normal prostate tissue | 160 |

**FDA-805-2**

| **position** | **no.** | **sex** | **age** | **organ** | **pathol. Diagnosis** | **BAIAP2L1 histoscore** |
| --- | --- | --- | --- | --- | --- | --- |
| A1 | 1 | F | 40 | Endometrium | Normal endometrium tissue | 0 |
| A2 | 2 | F | 21 | Endometrium | Normal endometrium tissue | 180 |
| A3 | 3 | F | 18 | Endometrium | Normal endometrium tissue | 10 |
| A4 | 4 | F | 48 | Uterine cervix | Normal uterine cervix tissue | 100 |
| A5 | 5 | F | 21 | Uterine cervix | Normal uterine cervix tissue | 100 |
| A6 | 6 | F | 35 | Uterine cervix | Normal uterine cervix tissue | 90 |
| A7 | 7 | M | 38 | Soft tissue | Normal skeletal muscle tissue | 0 |
| A8 | 8 | M | 30 | Soft tissue | Normal skeletal muscle tissue | 0 |
| A9 | 9 | M | 40 | Soft tissue | Normal skeletal muscle tissue | 0 |
| B1 | 10 | F | 42 | Skin | Normal skin tissue | 0 |
| B2 | 11 | M | 19 | Skin | Normal skin tissue | 140 |
| B3 | 12 | M | 30 | Skin | Normal skin tissue | 60 |
| B4 | 13 | F | 15 | Nerve | Normal nervous tissue (sparse) | 0 |
| B5 | 14 | F | 25 | Nerve | Normal nervous tissue | 0 |
| B6 | 15 | F | 62 | Nerve | Normal nervous tissue | 0 |
| B7 | 16 | M | 48 | Mesothelium | Normal mesothelium tissue (lung tissue) | 0 |
| B8 | 17 | M | 22 | Mesothelium | Normal mesothelial and lung tissue | 0 |
| B9 | 18 | M | 40 | Mesothelium | Normal mesothelium tissue (lung tissue) | 0 |
| C1 | 19 | M | 39 | Brain | Glioblastoma | 50 |
| C2 | 20 | M | 43 | Brain | Atypical meningioma | 0 |
| C3 | 21 | F | 15 | Brain | Malignant ependymoma | 70 |
| C4 | 22 | F | 61 | Brain | Malignant oligodendroglioma | 0 |
| C5 | 23 | F | 53 | Ovary | Serous papillary adenocarcinoma | 270 |
| C6 | 24 | F | 29 | Ovary | Mucinous papillary adenocarcinoma | 300 |
| C7 | 25 | F | 12 | Pancreas | Islet cell carcinoma | 0 |
| C8 | 26 | M | 64 | Pancreas | Adenocarcinoma | 180 |
| C9 | 27 | M | 32 | Testis | Seminoma | 140 |
| D1 | 28 | M | 30 | Testis | Embryonal carcinoma | 0 |
| D2 | 29 | F | 33 | Thyroid | Medullary carcinoma | 30 |
| D3 | 30 | M | 36 | Thyroid | Papillary carcinoma | 90 |
| D4 | 31 | F | 50 | Breast | Intraductal carcinoma | 0 |
| D5 | 32 | F | 62 | Breast | Lobular carcinoma in situ | 80 |
| D6 | 33 | F | 39 | Breast | Invasive ductal carcinoma | 90 |
| D7 | 34 | M | 21 | Spleen | Diffuse B-cell lymphoma | 0 |
| D8 | 35 | F | 45 | Lung | Small cell undifferentiated carcinoma | 80 |
| D9 | 36 | M | 74 | Lung | Squamous cell carcinoma | 100 |
| E1 | 37 | F | 59 | Lung | Adenocarcinoma | 80 |
| E2 | 38 | M | 50 | Esophagus | Squamous cell carcinoma | 300 |
| E3 | 39 | F | 67 | Esophagus | Adenocarcinoma | 180 |
| E4 | 40 | F | 73 | Stomach | Mucinous adenocarcinoma | 160 |
| E5 | 41 | M | 50 | Small intestine | Adenocarcinoma | 160 |
| E6 | 42 | F | 71 | Small intestine | malignant interstitialoma | 95 |
| E7 | 43 | M | 70 | Colon | Adenocarcinoma | 160 |
| E8 | 44 | M | 54 | Colon | malignant interstitialoma | 0 |
| E9 | 45 | F | 50 | Rectum | Adenocarcinoma | 100 |
| F1 | 46 | M | 32 | Rectum | malignant interstitialoma | 50 |
| F2 | 47 | M | 61 | Liver | Hepatocellular carcinoma | 10 |
| F3 | 48 | F | 17 | Liver | Hepatoblastoma | 50 |
| F4 | 49 | M | 55 | Kidney | Clear cell carcinoma | 140 |
| F5 | 50 | M | 67 | Prostate | Adenocarcinoma | 160 |
| F6 | 51 | M | 81 | Prostate | Transitional cell carcinoma | 300 |
| F7 | 52 | F | 45 | Endometrium | Leiomyoma | 0 |
| F8 | 53 | F | 51 | Endometrium | Endometrial adenocarcinoma | 160 |
| F9 | 54 | F | 39 | Endometrium | Clear cell | 150 |
| G1 | 55 | F | 36 | Uterine cervix | Squamous cell carcinoma | 160 |
| G2 | 56 | F | 41 | Uterine cervix | Squamous cell carcinoma | 50 |
| G3 | 57 | F | 20 | Soft tissue | Embryonal rhabdomyosarcoma of left leg | 60 |
| G4 | 58 | F | 70 | Rectum | Malignant melanoma of anus | 0 |
| G5 | 59 | F | 65 | Skin | Basal cell carcinoma of head | 30 |
| G6 | 60 | M | 46 | Skin | Squamous cell carcinoma (chest wall) | 285 |
| G7 | 61 | M | 59 | Mediastinum | Neurofibroma | 0 |
| G8 | 62 | F | 3 | Retroperitoneum | Neuroblastoma of retroperitoneum | 0 |
| G9 | 63 | M | 60 | Peritoneum | Malignant mesothelioma | 270 |
| H1 | 64 | M | 42 | Lymph node | Diffuse malignant lymphoma | 20 |
| H2 | 65 | F | 50 | Lymph node | Diffuse malignant B cell lymphoma (right thigh) | 0 |
| H3 | 66 | M | 53 | Lymph node | Hodgkin's lymphoma | 0 |
| H4 | 67 | F | 68 | Lymph node | Diffuse malignant lymphoma of mandible | 0 |
| H5 | 68 | F | 72 | Bladder | Transitional cell carcinoma | 160 |
| H6 | 69 | M | 62 | Bladder | Low grade malignant leiomyosarcoma | 0 |
| H7 | 70 | F | 7 | Bone | Osteosarcoma of right femur | 0 |
| H8 | 71 | F | 48 | Retroperitoneum | Spindle cell rhabdomyosarcoma | 0 |
| H9 | 72 | F | 60 | Soft tissue | Intermediate grade malignant leiomyosarcoma of left buttock | 0 |
