# Supplementary material for: BAI1-Associated Protein 2-Like 1 (BAIAP2L1) Is a Potential Biomarker in Ovarian Cancer
Source: PLoS One. 2015 Jul 29;10(7):e0133081. doi: 10.1371/journal.pone.0133081 (PMC4519316; doi:10.1371/journal.pone.0133081)
Supplement: S2 Tables — (DOCX) [file pone.0133081.s004.docx]

**S2 Tables. Tissue arrays (BC 111109, BC 111110, and OV8011-2-BX) and histoscores of BAIAP2L1**

**BC111109 ovarian cancer tissue arrays**

| **position** | **no.** | **age** | **pathol. Diagnosis** | **grade** | **stage** | **TNM** | **BAIAP2L1 histoscore** |
| --- | --- | --- | --- | --- | --- | --- | --- |
| G1 | 61 | 39 | Cancer adjacent normal ovary tissue | – | – | – | 0 |
| G2 | 62 | 53 | Cancer adjacent normal ovary tissue | – | – | – | 0 |
| G3 | 63 | 30 | Cancer adjacent normal ovary tissue | – | – | – | 0 |
| G4 | 64 | 39 | Cancer adjacent normal ovary tissue | – | – | – | 0 |
| G5 | 65 | 17 | Cancer adjacent normal ovary tissue | – | – | – | 0 |
| G6 | 66 | 40 | Cancer adjacent normal ovary tissue | – | – | – | 0 |
| G7 | 67 | 45 | Cancer adjacent normal ovary tissue | – | – | – | 0 |
| G8 | 68 | 42 | Cancer adjacent normal ovary tissue | – | – | – | 0 |
| G9 | 69 | 41 | Cancer adjacent normal ovary tissue | – | – | – | 0 |
| G10 | 70 | 69 | Cancer adjacent normal ovary tissue | – | – | – | 0 |
| F4 | 54 | 47 | Endometrioid adenocarcinoma | 1 | IIa | T2aN0M0 | 90 |
| F5 | 55 | 54 | Endometrioid adenocarcinoma | 1 | Ib | T1bN0M0 | 60 |
| F6 | 56 | 46 | Endometrioid adenocarcinoma | 1 | II | T2N0M0 | 90 |
| F7 | 57 | 49 | Endometrioid adenocarcinoma | 1 | I | T1N0M0 | 80 |
| F8 | 58 | 43 | Endometrioid adenocarcinoma | 2 | Ic | T1cN0M0 | 80 |
| F9 | 59 | 60 | Endometrioid adenocarcinoma | 3 | Ib | T1bN0M0 | 95 |
| F10 | 60 | 53 | Endometrioid adenocarcinoma | 3 | IIa | T2aN0M0 | 90 |
| E8 | 48 | 49 | Mucinous adenocarcinoma | 1 | Ia | T1aN0M0 | 95 |
| E9 | 49 | 45 | Mucinous adenocarcinoma | 1 | Ia | T1aN0M0 | 60 |
| E10 | 50 | 48 | Mucinous adenocarcinoma | 1 | Ib | T1bN0M0 | 120 |
| F1 | 51 | 41 | Mucinous adenocarcinoma | 3 | Ib | T1bN0M0 | 80 |
| F2 | 52 | 29 | Mucinous adenocarcinoma | 2 | III | T3N0M0 | 180 |
| F3 | 53 | 60 | Mucinous adenocarcinoma | 3 | Ia | T1aN0M0 | 95 |
| A7 | 7 | 39 | Serous adenocarcinoma | 1 | Ia | T1aN0M0 | 90 |
| A9 | 9 | 40 | Serous adenocarcinoma | 3 | IIa | T2aN0M0 | 100 |
| A10 | 10 | 54 | Serous adenocarcinoma | 2 | IIa | T2aN0M0 | 160 |
| D2 | 32 | 69 | Serous adenocarcinoma | 2 | II | T2N0M0 | 50 |
| A8 | 8 | 53 | Serous adenocarcinoma with necrosis | 3 | Ia | T1aN0M0 | 30 |
| A1 | 1 | 26 | Serous papillary adenocarcinoma | 1 | Ic | T1cN0M0 | 160 |
| A2 | 2 | 34 | Serous papillary adenocarcinoma | 1 | Ia | T1aN0M0 | 140 |
| A3 | 3 | 50 | Serous papillary adenocarcinoma | 1 | IIa | T2aN0M0 | 50 |
| A4 | 4 | 58 | Serous papillary adenocarcinoma | 2 | Ia | T1aN0M0 | 80 |
| A5 | 5 | 65 | Serous papillary adenocarcinoma | 1 | IIa | T2aN0M0 | 0 |
| A6 | 6 | 65 | Serous papillary adenocarcinoma | 1 | Ia | T1aN0M0 | 100 |
| B1 | 11 | 37 | Serous papillary adenocarcinoma | 2 | Ib | T1bN0M0 | 80 |
| B2 | 12 | 68 | Serous papillary adenocarcinoma | 2 | Ia | T1aN0M0 | 80 |
| B3 | 13 | 36 | Serous papillary adenocarcinoma | 3 | Ib | T1bN0M0 | 90 |
| B4 | 14 | 50 | Serous papillary adenocarcinoma | 3 | Ib | T1bN0M0 | 90 |
| B5 | 15 | 59 | Serous papillary adenocarcinoma | 3 | Ia | T1aN0M0 | 90 |
| B6 | 16 | 61 | Serous papillary adenocarcinoma | 2 | Ib | T1bN0M0 | 90 |
| B7 | 17 | 60 | Serous papillary adenocarcinoma | 2 | Ib | T1bN0M0 | 30 |
| B8 | 18 | 45 | Serous papillary adenocarcinoma | 2 | IIIc | T2N1M0 | 90 |
| B9 | 19 | 27 | Serous papillary adenocarcinoma | 2 | IIb | T2bN0M0 | 80 |
| B10 | 20 | 53 | Serous papillary adenocarcinoma | 3 | I | T1N0M0 | 70 |
| C1 | 21 | 54 | Serous papillary adenocarcinoma | 2 | Ib | T1bN0M0 | 30 |
| C2 | 22 | 52 | Serous papillary adenocarcinoma | 2 | Ia | T1aN0M0 | 50 |
| C3 | 23 | 64 | Serous papillary adenocarcinoma | 2 | IV | T3N1M1 | 30 |
| C4 | 24 | 58 | Serous papillary adenocarcinoma | 2 | IIb | T2bN0M0 | 80 |
| C5 | 25 | 51 | Serous papillary adenocarcinoma | 2 | Ia | T1aN0M0 | 90 |
| C6 | 26 | 66 | Serous papillary adenocarcinoma | 2 | Ic | T1cN0M0 | 10 |
| C7 | 27 | 47 | Serous papillary adenocarcinoma | 2 | IIa | T2aN0M0 | 50 |
| C8 | 28 | 57 | Serous papillary adenocarcinoma | 2 | IIa | T2aN0M0 | 20 |
| C9 | 29 | 58 | Serous papillary adenocarcinoma | 2 | IIIc | T3cN1M0 | 10 |
| C10 | 30 | 48 | Serous papillary adenocarcinoma | 2 | Ia | T1aN0M0 | 40 |
| D1 | 31 | 63 | Serous papillary adenocarcinoma | 2 | Ib | T1bN0M0 | 0 |
| D3 | 33 | 52 | Serous papillary adenocarcinoma | 3 | II | T2N0M0 | 80 |
| D4 | 34 | 58 | Serous papillary adenocarcinoma | 2 | I | T1N0M0 | 80 |
| D5 | 35 | 57 | Serous papillary adenocarcinoma | 2 | IIb | T2bN0M0 | 140 |
| D6 | 36 | 51 | Serous papillary adenocarcinoma | 2 | Ib | T1bN0M0 | 60 |
| D7 | 37 | 41 | Serous papillary adenocarcinoma | 3 | I | T1N0M0 | 60 |
| D8 | 38 | 52 | Serous papillary adenocarcinoma | 3 | Ic | T1cN0M0 | 190 |
| D9 | 39 | 41 | Serous papillary adenocarcinoma | 3 | Ia | T1aN0M0 | 180 |
| D10 | 40 | 60 | Serous papillary adenocarcinoma | 3 | Ia | T1aN0M0 | 80 |
| E1 | 41 | 70 | Serous papillary adenocarcinoma | 3 | I | T1N0M0 | 90 |
| E2 | 42 | 51 | Serous papillary adenocarcinoma | 3 | Ic | T1cN0M0 | 90 |
| E3 | 43 | 53 | Serous papillary adenocarcinoma | 3 | IIa | T2aN0M0 | 180 |
| E4 | 44 | 62 | Serous papillary adenocarcinoma | 3 | Ib | T1bN0M0 | 80 |
| E5 | 45 | 48 | Serous papillary adenocarcinoma | 3 | IIa | T2aN0M0 | 70 |
| E6 | 46 | 59 | Serous papillary adenocarcinoma | 2 | IIb | T2bN0M0 | 80 |
| E7 | 47 | 44 | Serous papillary adenocarcinoma | 3 | IIa | T2aN0M0 | 80 |

**BC111110 ovarian cancer tissue arrays**

| **position** | **no.** | **age** | **pathol. Diagnosis** | **grade** | **stage** | **TNM** | **BAIAP2L1 histoscore** |
| --- | --- | --- | --- | --- | --- | --- | --- |
| A1 | 1 | 46 | Serous papillary adenocarcinoma | 1 | IIb | T2bN0M0 | 30 |
| A2 | 2 | 60 | Serous papillary adenocarcinoma | 1 | IIa | T2aN0M0 | 70 |
| A3 | 3 | 45 | Serous papillary adenocarcinoma | 1 | Ia | T1aN0M0 | 90 |
| A4 | 4 | 70 | Serous papillary adenocarcinoma | 1 | Ia | T1aN0M0 | 80 |
| A5 | 5 | 39 | Serous papillary adenocarcinoma | 2 | Ia | T1aN0M0 | 90 |
| A6 | 6 | 53 | Serous papillary adenocarcinoma | 1 | Ia | T1aN0M0 | 90 |
| A7 | 7 | 49 | Serous papillary adenocarcinoma | 2 | Ia | T1aN0M0 | 70 |
| A8 | 8 | 73 | Serous papillary adenocarcinoma | 3 | Ib | T1bN0M0 | 90 |
| A9 | 9 | 57 | Serous papillary adenocarcinoma (fibrous tissue and blood vessel) | – | Ic | T1cN0M0 | 0 |
| A10 | 10 | 45 | Serous papillary adenocarcinoma | 2 | Ia | T1aN0M0 | 80 |
| B1 | 11 | 52 | Serous papillary adenocarcinoma | 2 | IIIc | T2N1M0 | 80 |
| B2 | 12 | 48 | Serous papillary adenocarcinoma | 2 | IIIc | T3cN0M0 | 90 |
| B3 | 13 | 48 | Serous papillary adenocarcinoma | 3 | Ib | T1bN0M0 | 95 |
| B4 | 14 | 47 | Serous papillary adenocarcinoma | 3 | I | T1N0M0 | 0 |
| B5 | 15 | 51 | Serous papillary adenocarcinoma | 3 | IIa | T2aN0M0 | 95 |
| B6 | 16 | 64 | Serous papillary adenocarcinoma | 3 | Ia | T1aN0M0 | 50 |
| B7 | 17 | 49 | Serous papillary adenocarcinoma | 2 | IIIc | T3cN0M0 | 95 |
| B8 | 18 | 51 | Serous papillary adenocarcinoma | 2 | IIIc | T3N1M0 | 90 |
| B9 | 19 | 51 | Serous papillary adenocarcinoma | 2 | Ia | T1aN0M0 | 50 |
| B10 | 20 | 65 | Serous papillary adenocarcinoma | 2 | IV | T2aN0M1 | 50 |
| C1 | 21 | 73 | Serous papillary adenocarcinoma | 3 | IIa | T2aN0M0 | 90 |
| C2 | 22 | 54 | Serous adenocarcinoma (sparse) | 2 | IIIb | T3bN0M0 | 80 |
| C3 | 23 | 52 | Serous papillary adenocarcinoma | 3 | IIa | T2aN0M0 | 10 |
| C4 | 24 | 51 | Serous papillary adenocarcinoma | 3 | Ia | T1aN0M0 | 80 |
| C5 | 25 | 52 | Serous papillary adenocarcinoma | 3 | Ia | T1aN0M0 | 160 |
| C6 | 26 | 52 | Serous papillary adenocarcinoma | 3 | IIIc | T3cN1M0 | 30 |
| C7 | 27 | 62 | Serous papillary adenocarcinoma | 3 | Ia | T1aN0M0 | 95 |
| C8 | 28 | 48 | Serous papillary adenocarcinoma | 2 | Ia | T1aN0M0 | 95 |
| C9 | 29 | 39 | Serous papillary adenocarcinoma with necrosis | 3 | II | T2N0M0 | 70 |
| C10 | 30 | 49 | Serous papillary adenocarcinoma | 3 | Ia | T1aN0M0 | 0 |
| D1 | 31 | 55 | Serous papillary adenocarcinoma | 2 | IV | T3cN1M1 | 80 |
| D2 | 32 | 48 | Serous papillary adenocarcinoma | 3 | Ib | T1bN0M0 | 120 |
| D3 | 33 | 51 | Serous papillary adenocarcinoma | 3 | Ib | T1bN0M0 | 70 |
| D4 | 34 | 56 | Serous papillary adenocarcinoma | 2 | IIIc | T3N1M0 | 95 |
| D5 | 35 | 60 | Serous papillary adenocarcinoma | 3 | Ib | T1bN0M0 | 100 |
| D6 | 36 | 41 | Serous papillary adenocarcinoma | 3 | IIa | T2aN0M0 | 100 |
| D7 | 37 | 39 | Serous papillary adenocarcinoma | 3 | Ib | T1bN0M0 | 95 |
| D8 | 38 | 32 | Serous papillary adenocarcinoma | 3 | IIIc | T3cN0M0 | 100 |
| D9 | 39 | 54 | Serous papillary adenocarcinoma | 3 | Ia | T1aN0M0 | 80 |
| D10 | 40 | 59 | Serous papillary adenocarcinoma | 3 | II | T2N0M0 | 80 |
| E1 | 41 | 50 | Serous papillary adenocarcinoma | 3 | Ib | T1bN0M0 | 80 |
| E2 | 42 | 46 | Mucinous papillary adenocarcinoma | 1 | Ia | T1aN0M0 | 100 |
| E3 | 43 | 48 | Mucinous papillary adenocarcinoma | 1 | Ib | T1bN0M0 | 80 |
| E4 | 44 | 39 | Mucinous papillary adenocarcinoma | 1 | Ia | T1aN0M0 | 90 |
| E5 | 45 | 55 | Mucinous adenocarcinoma | 1 | Ib | T1bN0M0 | 70 |
| E6 | 46 | 23 | Mucinous adenocarcinoma | 1 | Ic | T1cN0M0 | 80 |
| E7 | 47 | 58 | Mucinous papillary adenocarcinoma | 1 | Ia | T1aN0M0 | 90 |
| E8 | 48 | 55 | Mucinous papillary adenocarcinoma | 1 | Ib | T1bN0M0 | 80 |
| E9 | 49 | 60 | Mucinous papillary adenocarcinoma | 1 | Ia | T1aN0M0 | 60 |
| E10 | 50 | 19 | Mucinous papillary adenocarcinoma | 1 | IIa | T2aN0M0 | 60 |
| F1 | 51 | 29 | Mucinous papillary adenocarcinoma | 2 | IIa | T2aN0M0 | 80 |
| F2 | 52 | 46 | Mucinous papillary adenocarcinoma | 2 | IIa | T2aN0M0 | 80 |
| F3 | 53 | 37 | Mucinous papillary adenocarcinoma | 2 | Ia | T1aN0M0 | 90 |
| F4 | 54 | 39 | Mucinous papillary adenocarcinoma | 2 | Ib | T1bN0M0 | 70 |
| F5 | 55 | 37 | Mucinous adenocarcinoma | 3 | Ia | T1aN0M0 | 70 |
| F6 | 56 | 50 | Endometrioid adenocarcinoma | 1 | Ia | T1aN0M0 | 160 |
| F7 | 57 | 50 | Endometrioid adenocarcinoma | 1 | IIa | T2aN0M0 | 180 |
| F8 | 58 | 68 | Endometrioid adenocarcinoma | 2 | IIc | T2cN0M0 | 95 |
| F9 | 59 | 39 | Endometrioid adenocarcinoma | 3 | IIa | T2aN0M0 | 60 |
| G1 | 61 | 33 | Cancer adjacent normal ovary tissue | – | – | – | 0 |
| G2 | 62 | 59 | Cancer adjacent normal ovary tissue | – | – | – | 0 |
| G3 | 63 | 57 | Cancer adjacent normal ovary tissue | – | – | – | 0 |
| G4 | 64 | 41 | Cancer adjacent normal ovary tissue | – | – | – | 0 |
| G5 | 65 | 69 | Cancer adjacent normal ovary tissue | – | – | – | 0 |
| G6 | 66 | 49 | Cancer adjacent normal ovary tissue | – | – | – | 0 |
| G7 | 67 | 49 | Cancer adjacent normal ovary tissue | – | – | – | 0 |
| G8 | 68 | 50 | Cancer adjacent normal ovary tissue | – | – | – | 0 |
| G9 | 69 | 48 | Cancer adjacent normal ovary tissue | – | – | – | 0 |
| G10 | 70 | 42 | Cancer adjacent normal ovary tissue | – | – | – | 0 |

**OV8011-2-BX ovarian cancer tissue arrays**

| **position** | **no.** | **age** | **pathology** | **grade** | **stage** | **TNM** | **BAIAP2L1 histoscore** |
| --- | --- | --- | --- | --- | --- | --- | --- |
| A1 | 1 | 48 | Serous papillary adenocarcinoma | 2 | I | T1N0M0 | 300 |
| A2 | 2 | 38 | Serous papillary adenocarcinoma | 1 | IV | T3cN1M1 | 150 |
| A3 | 3 | 26 | Serous papillary adenocarcinoma | 1 | IC | T1cN0M0 | 180 |
| A4 | 4 | 34 | Serous papillary adenocarcinoma | 1 | IA | T1aN0M0 | 210 |
| A5 | 5 | 34 | Serous papillary adenocarcinoma | - | IB | T1bN0M0 | 90 |
| A6 | 6 | 58 | Serous papillary adenocarcinoma with necrosis | 2 | I | T1N0M0 | 120 |
| A7 | 7 | 33 | Serous papillary adenocarcinoma | 2 | I | T1N0M0 | 285 |
| A8 | 8 | 52 | Serous papillary adenocarcinoma | 2 | II | T2N0M0 | 50 |
| A9 | 9 | 55 | Serous papillary adenocarcinoma | 2 | I | T1N0M0 | 170 |
| A10 | 10 | 26 | Serous papillary adenocarcinoma | 2 | IIIC | T3cN1M0 | 300 |
| B1 | 11 | 35 | Serous papillary adenocarcinoma | 2 | IA | T1aN0M0 | 255 |
| B2 | 12 | 22 | Serous papillary adenocarcinoma | 2 | IIB | T2bN0M0 | 1 |
| B3 | 13 | 41 | Serous adenocarcinoma | 3 | IA | T1aN0M0 | 255 |
| B4 | 14 | 56 | Serous cystadenocarcinoma | 3 | IB | T1bN0M0 | 300 |
| B5 | 15 | 52 | Serous cystadenocarcinoma | 3 | IIA | T2aN0M0 | 1 |
| B6 | 16 | 38 | Serous papillary adenocarcinoma | 3 | IIIC | T3bN1M0 | 150 |
| B7 | 17 | 48 | Serous papillary adenocarcinoma | 3 | IIIC | T3cN0M0 | 1 |
| B8 | 18 | 65 | Serous papillary adenocarcinoma | 3 | IA | T1aN0M0 | 60 |
| B9 | 19 | 60 | Serous papillary adenocarcinoma | 3 | IB | T1bN0M0 | 10 |
| B10 | 20 | 50 | Serous papillary adenocarcinoma | 3 | IC | T1cN0M0 | 1 |
| C1 | 21 | 56 | Serous adenocarcinoma | 3 | IIIC | T2N1M0 | 140 |
| C2 | 22 | 35 | Serous papillary adenocarcinoma | 3 | I | T1N0M0 | 270 |
| C3 | 23 | 56 | Serous adenocarcinoma with necrosis | 3 | IV | T3cN1M1 | 0 |
| C4 | 24 | 63 | Serous adenocarcinoma with necrosis | 3 | II | T2N0M0 | 190 |
| C5 | 25 | 65 | Serous papillary adenocarcinoma | 3 | II | T2N0M0 | 140 |
| C6 | 26 | 51 | Serous adenocarcinoma | 3 | II | T2N0M0 | 285 |
| C8 | 28 | 50 | Serous adenocarcinoma | 3 | IA | T1aN0M0 | 60 |
| C9 | 29 | 70 | Serous adenocarcinoma | 3 | IA | T1aN0M0 | 50 |
| C10 | 30 | 48 | Serous adenocarcinoma | 3 | IIA | T2aN0M0 | 20 |
| D1 | 31 | 59 | Serous papillary adenocarcinoma | 3 | IIB | T2bN0M0 | 90 |
| D2 | 32 | 39 | Serous papillary adenocarcinoma | 3 | IB | T1bN0M0 | 30 |
| D3 | 33 | 60 | Serous adenocarcinoma | 3 | I | T1N0M0 | 285 |
| D4 | 34 | 40 | Serous adenocarcinoma (sparse) | 3 | I | T1N0M0 | 180 |
| D5 | 35 | 48 | Serous adenocarcinoma (sparse) | 3 | IV | T3N1M1 | 285 |
| D6 | 36 | 49 | Clear cell carcinoma | - | IA | T1aN0M0 | 297 |
| D7 | 37 | 43 | Clear cell carcinoma | - | IA | T1aN0M0 | 300 |
| D8 | 38 | 44 | Clear cell carcinoma with necrosis | - | IC | T1cN0M0 | 5 |
| D9 | 39 | 50 | Clear cell carcinoma with necrosis | - | I | T1N0M0 | 80 |
| D10 | 40 | 48 | Clear cell carcinoma | - | I | T1N0M0 | 270 |
| E1 | 41 | 53 | Clear cell carcinoma | - | I | T1N0M0 | 270 |
| E2 | 42 | 53 | Clear cell carcinoma | - | IB | T1bN0M0 | 20 |
| E3 | 43 | 51 | Clear cell carcinoma | - | IA | T1aN0M0 | 180 |
| E4 | 44 | 47 | Clear cell carcinoma | - | IIA | T2aN0M0 | 285 |
| E5 | 45 | 42 | Clear cell carcinoma | - | IA | T1aN0M0 | 285 |
| E6 | 46 | 61 | Clear cell carcinoma | - | IA | T1aN0M0 | 285 |
| E7 | 47 | 37 | Clear cell carcinoma | - | II | T2N0M0 | 0 |
| E8 | 48 | 66 | Clear cell carcinoma | - | IIA | T2aN0M0 | 120 |
| E9 | 49 | 50 | Clear cell carcinoma | - | I | T1N0M0 | 30 |
| E10 | 50 | 68 | Clear cell carcinoma | - | II | T2N0M0 | 10 |
| F1 | 51 | 47 | Endometrioid adenocarcinoma | 1 | IIA | T2aN0M0 | 30 |
| F2 | 52 | 46 | Endometrioid adenocarcinoma | 2 | II | T2N0M0 | 10 |
| F3 | 53 | 69 | Endometrioid adenocarcinoma | 1 | IA | T1aN0M0 | 60 |
| F4 | 54 | 50 | Endometrioid adenocarcinoma | 2 | IIA | T2aN0M0 | 240 |
| F5 | 55 | 49 | Endometrioid adenocarcinoma | 2 | I | T1N0M0 | 170 |
| F6 | 56 | 49 | Endometrioid adenocarcinoma | 1 | IA | T1aN0M0 | 40 |
| F7 | 57 | 45 | Endometrioid adenocarcinoma | 2 | IIA | T2aN0M0 | 140 |
| F8 | 58 | 43 | Endometrioid adenocarcinoma | - | IA | T1aN0M0 | 0 |
| F9 | 59 | 68 | Endometrioid adenocarcinoma | 3 | IIC | T2cN0M0 | 5 |
| F10 | 60 | 55 | Endometrioid adenocarcinoma | 3 | I | T1N0M0 | 10 |
| G1 | 61 | 51 | Endometrioid adenocarcinoma | 3 | I | T1N0M0 | 5 |
| G2 | 62 | 47 | Endometrioid adenocarcinoma with necrosis | 3 | IC | T1cN0M0 | 5 |
| G3 | 63 | 53 | Endometrioid adenocarcinoma | 3 | IIA | T2aN0M0 | 120 |
| G4 | 64 | 50 | Endometrioid adenocarcinoma | 3 | IIIC | T3bN1M0 | 75 |
| G5 | 65 | 60 | Endometrioid adenocarcinoma | 3 | IB | T1bN0M0 | 80 |
| G6 | 66 | 50 | Mucinous adenocarcinoma | 2 | IA | T1aN0M0 | 30 |
| G7 | 67 | 40 | Mucinous adenocarcinoma | 2 | IA | T1aN0M0 | 180 |
| G8 | 68 | 39 | Mucinous adenocarcinoma | 1 | IB | T1bN0M0 | 40 |
| G9 | 69 | 45 | Mucinous adenocarcinoma | 2 | IA | T1aN0M0 | 90 |
| G10 | 70 | 40 | Mucinous adenocarcinoma (sparse) | 3 | IC | T1cN0M0 | 30 |
| H1 | 71 | 52 | Mucinous adenocarcinoma (sparse) | 1 | I | T1N0M0 | 180 |
| H2 | 72 | 45 | Mucinous adenocarcinoma | 3 | IA | T1aN0M0 | 300 |
| H3 | 73 | 37 | Mucinous adenocarcinoma | 3 | I | T1N0M0 | 190 |
| H4 | 74 | 50 | Mucinous adenocarcinoma | 3 | I | T1N0M0 | 30 |
| H5 | 75 | 49 | Mucinous adenocarcinoma | 1 | IA | T1aN0M0 | 10 |
