# Supplementary material for: BAI1-Associated Protein 2-Like 1 (BAIAP2L1) Is a Potential Biomarker in Ovarian Cancer
Source: PLoS One. 2015 Jul 29;10(7):e0133081. doi: 10.1371/journal.pone.0133081 (PMC4519316; doi:10.1371/journal.pone.0133081)
Supplement: S3 Tables — (DOCX) [file pone.0133081.s005.docx]

**S3 Tables Clinical information of 14 cases of ovarian cancers and histoscores of BAIAP2L1 in primary cancer and metastasized sites**

| Ovca no | Patho Diagnosis | Metastatic site | FIGO stage | Grade | Age |
| --- | --- | --- | --- | --- | --- |
| OV- 0001 | Endometrioid carcinoma | omentum | IIIC | 3 | 72 |
| OV- 0008 | Serous adenocarcinoma | omentum | IV | 3 | 62 |
| OV- 0017 | Serous adenocarcinoma | brain | IV | 3 | 50 |
| OV- 0019 | Serous adenocarcinoma | Cecum | IIIC | 3 | 57 |
| OV- 0051 | Clear cell carcinoma | Lymph node | IIIC | 3 | 35 |
| OV- 0077 | Clear cell carcinoma | Omentum | IIIB | 3 | 58 |
| OV- 0101 | Mucinous adenocarcinoma | Omentum | IC | 1 | 72 |
| OV- 0122 | Serous papillary adenocarcinoma | Parametrium | IIC | 3 | 43 |
| OV- 0184 | Clear cell carcinoma | Lymph node | IIIC | 2 | 41 |
| OV- 0188 | Clear cell carcinoma | Omentum | IIIC | 3 | 54 |
| OV- 0195 | Mucinous adenocarcinoma | Omentum | IIIC | 1 | 49 |
| OV- 0211 | Endometrioid carcinoma | Omentum | IIIC | 3 | 42 |
| OV- 0225 | Endometrioid carcinoma | Small bowel | IIIC | 1 | 60 |
| OV- 0228 | Endometrioid carcinoma | Lymph node | IIIC | 3 | 60 |
